# Antimicrobial resistances do not affect colonization parameters of intestinal E. coli in a small piglet group

**DOI:** 10.1186/1757-4749-1-18

**Published:** 2009-10-08

**Authors:** Peter Schierack, Kristina Kadlec, Sebastian Guenther, Matthias Filter, Stefan Schwarz, Christa Ewers, Lothar H Wieler

**Affiliations:** 1 Institut für Mikrobiologie und Tierseuchen, Freie Universität Berlin, Philippstr 13, 10115 Berlin, Germany; 2 Fachbereich Bio-, Chemie- und Verfahrenstechnik, Hochschule Lausitz (FH), Großenhainer Straße 57, 01968 Senftenberg, Germany; 3 Institut für Nutztiergenetik, Friedrich-Loeffler-Institut (FLI), Bundesforschungsinstitut für Tiergesundheit, Höltystr 10, 31535 Neustadt-Mariensee, Germany; 4 Institut für Molekularbiologie und Bioinformatik, Charite-Universitätsmedizin Berlin, Arnimallee 22, 14195 Berlin, Germany

## Abstract

**Background:**

Although antimicrobial resistance and persistence of resistant bacteria in humans and animals are major health concerns worldwide, the impact of antimicrobial resistance on bacterial intestinal colonization in healthy domestic animals has only been rarely studied. We carried out a retrospective analysis of the antimicrobial susceptibility status and the presence of resistance genes in intestinal commensal *E. coli *clones from clinically healthy pigs from one production unit with particular focus on effects of pheno- and/or genotypic resistance on different nominal and numerical intestinal colonization parameters. In addition, we compared the occurrence of antimicrobial resistance phenotypes and genotypes with the occurrence of virulence associated genes typical for extraintestinal pathogenic *E. coli*.

**Results:**

In general, up to 72.1% of all *E. coli *clones were resistant to ampicillin, chloramphenicol, kanamycin, streptomycin, sulfamethoxazole or tetracycline with a variety of different resistance genes involved. There was no significant correlation between one of the nominal or numerical colonization parameters and the absence or presence of antimicrobial resistance properties or resistance genes. However, there were several statistically significant associations between the occurrence of single resistance genes and single virulence associated genes.

**Conclusion:**

The demonstrated resistance to the tested antibiotics might not play a dominant role for an intestinal colonization success in pigs in the absence of antimicrobial drugs, or cross-selection of other colonization factors e.g. virulence associated genes might compensate "the cost of antibiotic resistance". Nevertheless, resistant strains are not outcompeted by susceptible bacteria in the porcine intestine.

**Trial Registration:**

The study was approved by the local animal welfare committee of the "Landesamt für Arbeitsschutz, Gesundheitsschutz und technische Sicherheit" Berlin, Germany (No. G0037/02).

## Background

Antimicrobial resistant bacteria are recognized as a health problem world-wide. Application of antimicrobial agents enhanced not only the occurrence of antimicrobial resistance in pathogenic bacteria, but also in commensal bacteria which might serve as a reservoir of resistance genes for pathogenic bacteria [1-3]. The persistence of resistant bacteria in the absence of direct selective pressure by the use of antimicrobial agents is one of the most serious emerging health concerns.

It has been discussed that the existence of resistance charges the general metabolism of a bacterium ("the cost of antibiotic resistance"). Consequently, without a selective pressure of the respective antimicrobial agent, such resistant bacteria should be easily outcompeted by susceptible bacteria in their habitats [4]. However, resistant bacteria often persist over long periods in the absence of selective pressures. One explanation is the "adaptation of bacteria to the fitness costs of antibiotic resistance". Several authors demonstrated that bacteria can develop resistance which gives the resistant bacterium even a colonization advantage compared to susceptible bacteria [5,6]. A second explanation is a co-selection. Genes for antimicrobial resistance often occur on mobile genetic elements, such as plasmids, transposons or integrons. Such mobile genetic elements might carry more than one antimicrobial resistance gene as well as genes coding for virulence or colonization factors. A selective advantage of any gene located on the mobile genetic element might enhance the persistence of such strains [7]. A third explanation is that certain proteins e.g. multidrug transporters play an important role not only in antimicrobial resistance, but also in bacterial cell metabolism. Multidrug transporters commonly exhibit a wide substrate spectrum, which includes structurally and functionally unrelated substances such as antimicrobial agents, detergents or dyes, in addition to toxic products from cell metabolism [8,9]. Thus, the presence of multidrug transporters might help bacteria to successfully colonize the intestine.

The dynamics of antibiotic resistance in commensals has not been studied to a great extent [10]. For example, it is not known whether antibiotic resistance influences the capacity of a given strain to persist in the normal microbiota of a host. Almost all data concerning competition advantages/disadvantages of resistant to sensitive bacteria were derived using culture medium in test tubes or using mouse models with only single or very few bacterial strains included [11,12]. Just recently, Karami et al. described effects of antimicrobial resistance to tetracycline and ampicillin on competition advantages/disadvantages of resistant to sensitive bacteria in the human intestine during one single study [10,13]. However, to our best knowledge data related to domestic animals have not been published yet.

*E. coli *are indicator bacteria representing the Gram negative bacterial microflora. These autochthonous bacteria are commonly isolated from animal and human feces, they are relevant to animal and human medicine and many resistance phenotypes present in bacteria from animals are present in this species [14]. Intestinal *E. coli *are also a part of the autochthonous porcine intestinal microflora and the widespread occurrence of antimicrobial resistance in pathogenic as well as commensal porcine *E. coli *is connected to a number of different resistance gene profiles [15]. Conclusively, porcine commensal *E. coli *seemed to be a good model to study whether antimicrobial resistance might promote or reduce the colonization success of intestinal *E. coli*.

Therefore, we compared in a retrospective analysis the detection frequency of *E. coli *clones in one sow and five of her piglets with their antimicrobial resistance pheno- and genotypes. The study started with the birth of the piglets and was completed at the piglets age of 56 days which was 28 days after weaning. Thus, we were able to monitor correlations between antimicrobial susceptibilities and the presence of resistance genes with (i) the transmission of *E. coli *from the sow to here piglets, (ii) the colonization of the fast developing intestine of young piglets and (iii) weaning, an event which represents a situation of severe changes of the intestinal milieu of the piglets. Additionally, we compared the occurrence of single resistance genes with the occurrence of virulence associated genes (VAGs) typical for extraintestinal pathogenic *E. coli *(ExPEC) which might also play a role for intestinal colonization [16,17]. The study also included the initial comprehensive description of the antimicrobial resistance status of the commensal porcine intestinal *E. coli *microflora of a single small pig production unit and the identification of the resistance genes involved.

## Methods

### Animal housing

The pig production unit consisted of approximately 40 sows and their piglets and was located in Berlin, Germany. Pigs (hybrids of Deutsche Landrasse and Duroc) were stalled in groups and fed according to the National Research Council recommendations [18]. The basal diet for sows and piglets mainly comprised barley and wheat, and wheat and soybean meal respectively. Sows were fed restrictively according to their body mass and litter size. Piglets had *ad libitum *access to feed. Sows and piglets had *ad libitum *access to water. The administration of antimicrobial substances was prohibited for both sows and piglets for at least 3 months prior to the Resistance Status Study trial. The sow of the Colonization Study received no antimicrobial substances 14 months prior to the trial except of two applications of Cefquinome (Cobactan^®^, Veterinaria AG, Zürich, Switzerland) 10 months prior to the trial. The piglets of the Colonization Study received no antimicrobial substances during the examinations. Piglets were weaned at day 28 of age. Piglets were reared after weaning in segregated pens of flat-deck batteries. The health status of the pigs was monitored by control of the general animal condition and fecal consistency. The study was approved by the local animal welfare committee of the "Landesamt für Arbeitsschutz, Gesundheitsschutz und technische Sicherheit" Berlin, Germany (No. G0037/02).

### Isolation of *E. coli *for the determination of the antimicrobial resistance status (Resistance Status Study)

To determine the general antimicrobial resistance status of intestinal *E. coli *from the pig production unit, *E. coli *were isolated from 15 clinically healthy piglets (age 56 days) from 12 different sows. This included the isolation from digesta as well as mucosa samples from the jejunum and colon. *E. coli*-like isolates [pink colonies on CHROM agar orientation plates [19], purple on MacConkey, blue or green on GASSNER agar plates] were assigned to clones by macrorestriction analysis (see below). Clones were finally identified as *E. coli *using standard methods [20]. The detailed isolation protocol has recently been published [21]. Forty nine isolates, each representing one *E. coli *clone, were further tested for antimicrobial susceptibility and resistance genes as mentioned below. These *E. coli *clones comprised dominant as well as minor clones [22] from the jejunum as well as colon.

### Isolation of *E. coli *for analysis of effects of resistance phenotypes and genotypes on an intestinal colonization success (Colonization Study)

To compare the intestinal colonization success of resistant and susceptible *E. coli*, the transfer of *E. coli *from one sow to five of her piglets was studied. The sow and the five piglets were from the same pig production unit like the piglets of the Resistance Status Study. The Colonization Study was carried out over a period of eight weeks, with samples being taken weekly. Sampling was started with sow rectal content taken on the day of birth of the piglets; sampling from the five piglets was started one week later (when piglets were seven days old); sow feces were sampled one week prior to weaning (when piglets were 21 days old); and the last piglet samples were taken at the age of 56 days. Additionally, teats and the bottom of the box were wiped off with sterile cotton swabs. In summary, a total of 1200 *E. coli *isolates had been isolated: 80 rectal isolates from one sow (over a period of four weeks), 80 isolates from the teats of the sow (four weeks), 80 isolates from the bottom of the sow box (four weeks) and 160 isolates from each of five piglets (eight weeks). Before the piglets were transported to their separation box after weaning, the bottom of the box was wiped off with sterile cotton swabs. Serial dilutions of samples were plated on MacConkey (Oxoid, Hampshire, UK) agar plates and incubated for 18 h at 37°C. Twenty colonies per sample and animal were randomly chosen and plated on GASSNER (Sifin, Berlin, Germany) and CHROM agar orientation (Chromagar, Paris, France) [19] plates. *Enterobacteriaceae*-like colonies (purple on MacConkey, blue or green on GASSNER and pink color on CHROM agar orientation plates) were pre-defined as *E. coli*.

Twenty *E. coli *colonies per sample were differentiated by macrorestriction analysis (PFGE) according to Hartley et al. (1979) who found that results were similar if they picked 20 colonies compared to 100 colonies to isolate both the predominant *E. coli *type and most of the minor types [23]. PFGE was performed as previously described [24]. Bacterial DNA was digested with 20 U *Xba*I (Promega, WI, USA) at 37°C overnight. DNA fragments were separated in a 1.2% pulsed-field agarose gel for 22 h at 6V, pulse 5-50. Evaluation of PFGE profiles for similarity was performed using Bionumerics software with the UPGMA method (unweighted pair group method with arithmetic mean) and Dice similarity indices (complete linkage; optimization, 1%; position tolerance, 1.3%; Applied Maths, Belgium). After macrorestriction analysis, each determined clone was additionally verified as *E. coli *using standard methods [20].

### Antimicrobial susceptibility testing

For the determination of the general antimicrobial resistance status of *E. coli *from the pig population (Resistance Status Study), 49 *E. coli *strains from domestic piglets were tested for susceptibility to the following antimicrobial agents by the microdilution broth method as recommended by the Clinical and Laboratory Standards Institute [25] (breakpoints for resistance are indicated in parentheses): ampicillin (≥32 μg/ml), amoxicillin/clavulanic acid (≥32/16 μg/ml), cephalothin (≥32 μg/ml), cefazolin (≥32 μg/ml), chloramphenicol (≥32 μg/ml), enrofloxacin (no breakpoint available), gentamicin (≥16 μg/ml), kanamycin (≥32 μg/ml), neomycin (≥32 μg/ml, [26]), spectinomycin (no breakpoint available), streptomycin (no breakpoint available), tetracycline (≥16 μg/ml) and sulfamethoxazole (≥512 μg/ml). Although there were no breakpoints available for spectinomycin and streptomycin for further analysis we defined *E. coli *resistant to these antimicrobial agents if the MIC of spectinomycin to this *E. coli *isolate was ≥128 μg/ml and to streptomycin ≥64 μg/ml. We assumed that these concentrations can not be clinically achieved in pigs. To perform the tests, commercially acquired microtiter plates (Sensititre, MCS Diagnostics, UK) were used.

For the Colonization Study one representative isolate of each *E. coli *clone from the sow and five of her piglets was tested for antimicrobial resistance by disk diffusion according to the Clinical and Laboratory Standards Institute [25]. Only the antimicrobial agents were included for which resistance was detected during the Resistance Status Study: ampicillin, chloramphenicol, kanamycin, neomycin, spectinomycin, streptomycin, tetracycline, sulfamethoxazole.

### Resistance gene determination using PCR

Resistant *E. coli *strains were tested via PCR for the presence of the respective resistance genes *bla*_TEM _(resistance to ampicillin), *catA1 *and *cmlA/B *(resistance to chloramphenicol), *aph(3')-Ia *(resistance to kanamycin and neomycin), *aadA *(resistance to streptomycin-spectinomycin), *strA/strB *(resistance to streptomycin), *tet*(A), *tet*(B), *tet*(C) (resistance to tetracycline) as well as *sul1*, *sul2 *and *sul3 *(resistance to sulfonamides). Primers and PCR reaction conditions were performed as previously described [15,27-32].

### Determinations of virulence-associated genes (VAGs) typical for extraintestinal pathogenic *E. coli *(ExPEC) using PCR

Tested VAGs are listed in Table 1. Primers and PCR conditions were previously described [17,33].

**Table 1:** Tested extraintestinal *E. coli *(ExPEC)-typical virulence associated genes (VAGs).

**Gene(s) or operon**	**Description**
Adhesins	

*afa/draB*	Afimbrial/Dr antigen-specific adhesin
*crl*	Curli fiber gene
*fimC*	Type 1 fimbriae (D-mannose specific adhesin)
*hra*	Heat-resistant agglutinin
*iha*	Iron-regulated-gene-homologue adhesin
*papC*	Pilus associated with pyelonephritis
*sfa/focCD*	S fimbriae (sialic acid-specific) and F1C fimbriae
*tsh*^1^	Temperature sensitive hemagglutinin
*mat*	Meningitis associated and temperature regulated fimbriae

Iron acquisition	

*chuA*	Heme receptor gene (*E. coli *haem utilization)
*fyuA*	Ferric yersinia uptake (yersiniabactin receptor)
*ireA*	Iron-responsive element (putative catecholate siderophore receptor)
*iroN*^1^	Catecholate siderophore (salmochelin) receptor
*irp2*	Iron repressible protein (yersiniabactin synthesis)
*iucD*^1^	Aerobactin synthesis
*sitD *chr.	*Salmonella *iron transport system gene
*sitD *ep.^1^	*Salmonella *iron transport system gene

Protectins/Serum resistance	

*cvi/cva*^1^	Structural genes of colicin V operon (Microcin ColV)
*iss*^1^	Increased serum survival
*neuC*	K1 capsular polysaccharide
*kpsMT *II	Group II capsule antigens
*ompA*	Outer membrane protein
*traT*^1^	Transfer Protein

Toxins	

*astA*	EAST1 (heat stable cytotoxin associated with enteroaggregative *E. coli*)
*sat*	Secreted autotransporter toxin
*vat*	Vacuolating autotransporter toxin
*hlyA*	Haemolysin A

Invasins	

*gimB*	Genetic island associated with newborn meningitis
*ibeA*	Invasion of brain endothelium
*tia*	Toxigenic invasion locus in ETEC strains

Miscellaneous	

*pic*	Serin protease autotransporter
*malX*	Pathogenicity-associated island marker CFT073

^1 ^Genes associated with large plasmids in APEC, like pAPEC-O2-ColV [NC_007675], pTJ100 [AY553855], and pAPEC-O1-ColBM [DQ381420].

Primer references are cited in Ewers et al[33].

### Definitions and statistical analysis

As we sampled all isolates during a short period of time from rectal contents of animals of one small, well-defined pig production unit, *E. coli *isolates which showed in their PFGE patterns not more than one band difference were defined as members of the same clone [34]. We defined a solitary clone of the sow as a clone found only in one rectal sample from the sow. Consequently, we defined a solitary clone of one piglet as a clone found only in one rectal sample from this piglet. A dominant clone was defined as a clone which represented ≥ 50% of typed colonies in one sample, a minor clone was defined as a clone which represented ≤ 10% of typed colonies in one sample according to Schlager et al[22]. The statistical analysis was performed using the software SPSS 12.0 (SPSS Inc., Chicago, IL, U.S.A.). Hypotheses on associations between nominal parameters (antibiotic resistance, resistance genes, VAGs) were tested by application of Fisher's exact test. Hypotheses on relationships between the number of resistance phenotypes and genotypes and the number of VAGs were tested by analyzing the non-parametric Spearman rank correlation coefficient. Relationships between nominal and numerical parameters were tested by application of the Mann-Whitney-U test. P-values below an alpha = 0.05 were considered significant.

## Results

### Occurrence of resistance phenotypes and genotypes in *E. coli *clones in one pig population (Resistance Status Study)

All animals were clinically healthy which was proved by monitoring the general animal condition and the fecal consistency. To define existing antimicrobial resistance, 49 *E. coli *clones from 15 piglets were tested to 15 antimicrobial agents by the broth microdilution method. Thirty-three clones (67.4% of clones) were resistant to at least one antimicrobial agent, with resistance to ampicillin (20.4%), chloramphenicol (2.0%), kanamycin (10.2%), neomycin (10.2%), spectinomycin (34.7%), streptomycin (51.0%), tetracycline (65.3%) and sulfamethoxazole (36.7%). A total of 13 different resistance profiles were detectable with six clones (12.2%) being resistant to one antimicrobial agent, five clones (10.2%) being resistant to two antimicrobial agents, five clones (10.2%) being resistant to three antimicrobial agents, eight clones (16.3%) resistant to four antimicrobial agents, six clones (12.2%) being resistant to five antimicrobial agents, one clone (2.0%) resistant to six antimicrobial agents, and two clones (4.1%) being resistant to seven antimicrobial agents. Sixteen clones (32.6%) were susceptible to all tested antimicrobial substances. Resistant *E. coli *carried the resistance genes *bla*_TEM_, *catA1, aph(3')-I*, *aadA*, *strA*, *strB*, *tet*(A), *tet*(B), *sul1*, *sul2*, and/or *sul3*. In a single sulfonamide resistant isolate no gene coding for resistance to sulfamethoxazole was detected. Up to seven different resistance genes in one strain and a total of 18 different resistance gene profiles were detectable (see Additional file 1).

### Occurrence of resistance phenotypes and genotypes in *E. coli *clones from one sow and five of her piglets (Colonization Study)

All animals remained clinically healthy throughout the Colonization Study which was proved by monitoring the general animal condition and fecal consistency. During the Colonization Study a total of 1200 *E. coli *isolates had been affiliated to clones via macrorestriction analysis. *E. coli *were not detectable in feed and drinking water.

A total of 44 clones were isolated from the five piglets, 34 clones from the sow rectal content, 28 clones from the teats and 23 clones from the bottom of the sow box. PFGE patterns varied considerably between single *E. coli *clones and indicated that clones were unrelated [34]. These results have recently already been published in more detail [17]. With one exception, all isolates of one clone did not differ in their PFGE band pattern. In the one exceptional case there was one clone which comprised eight isolates (one isolate from the teats of the sow, six isolates from one piglet, one isolate from another piglet) one of which had an additional 97 kb fragment. Isolates of several clones were found in samples of different sampling sizes resulting in a total of 68 different *E. coli *clones.

**Figure 1:**
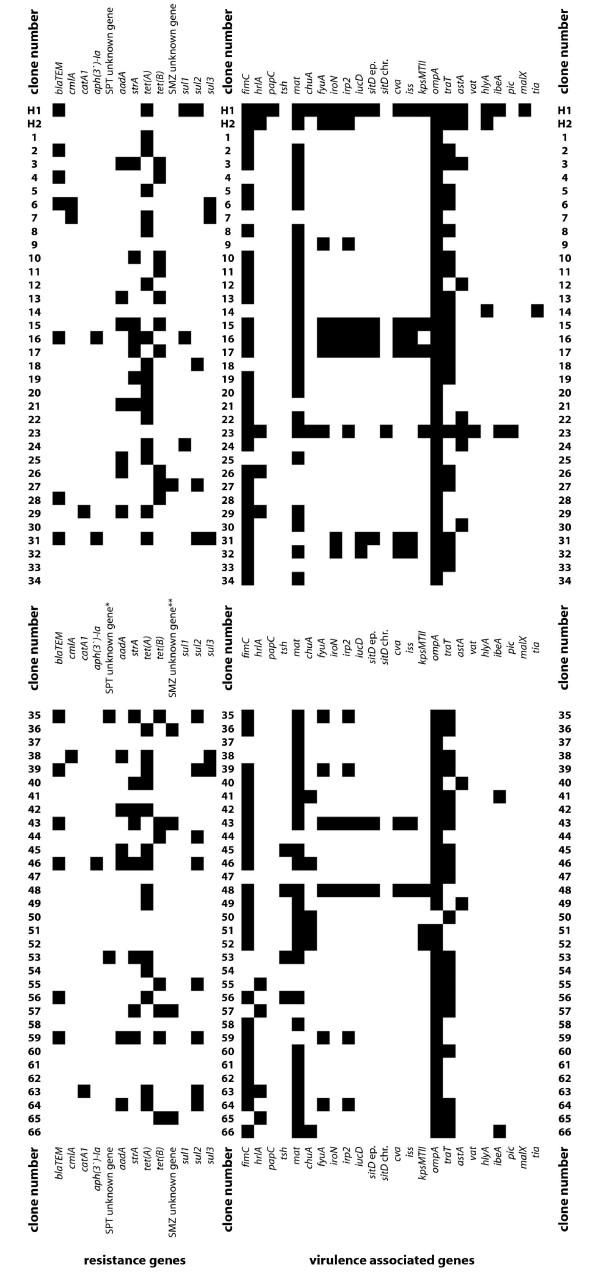
**Resistance (left rows) and ExPEC-typical virulence associated genes (right rows) of *E. coli *(Colonization Study)**. Genes never detected are not included in this figure. Additionally, *crlA *is not shown as all clones carried this gene. SPT: resistance to spectinomycin; SMZ: resistance to sulfamethoxazole. Clones H1 and H2 were hemolytic, clones 1 to 66 non-hemolytic.

All 68 *E. coli *clones were tested to the 8 antimicrobial agents against which there was resistance present in this pig population as revealed by our Resistance Status Study. Tests were done by the disk diffusion method [25]. Of the 68 clones, 49 clones (72.1% of all clones) were resistant to up to seven of the antimicrobial agents whereas 19 clones (27.9%) were susceptible to all tested antimicrobial agents. Clones were resistant to ampicillin (19.1%), chloramphenicol (7.4%), kanamycin (4.4%), neomycin (4.4%), streptomycin (32.4%), spectinomycin (20.6%), tetracycline (70.6%) and sulfamethoxazole (33.8%). A total of 17 different resistance profiles were detectable with 9 clones (13.2%) resistant to one antimicrobial agent, with 17 clones (25.0%) resistant to two antimicrobial agents, 13 clones (19.1%) resistant to three antimicrobial agents, four clones (5.9%) resistant to four antimicrobial agents, four clones (5.9%) resistant to five antimicrobial agents, one clone (1.5%) resistant to six antimicrobial agents and one clone (1.5%) resistant to seven antimicrobial agents (see Aditional file 2). Resistant *E. coli *carried the resistance genes *bla*_TEM_, *catA1, cmlA*, *aph(3')-I*, *aadA*, *strA*, *strB*, *tet*(A), *tet*(B), *sul1*, *sul2*, and/or *sul3*. No respective resistance gene was detected for two isolates resistant to sulfamethoxazole and one isolate resistant to spectinomycin. Up to seven different resistance genes in one strain and a total of 31 different resistance gene profiles were identified. The detected resistance genes are included in Figure 1 (see also Additional file 2).

### Analysis of correlations between antimicrobial resistance phenotypes and genotypes and colonization parameters (Colonization Study)

*E. coli *exclusively found in the sow (n = 17) were not significantly more or less resistant or carried significantly more or less resistance genes than *E. coli *exclusively isolated from piglets (n = 28). To test for the correlation between resistance phenotypes and genotypes and the ability of intestinal colonization of piglets, we included all 44 clones which were isolated from the piglets and analyzed the correlations between the sum of all resistance phenotypes and genotypes of each clone and numerical criteria describing colonization success. Additionally, effects of single resistance/resistance genes on nominal and numerical criteria relevant for colonization were tested. Nominal colonization criteria included the detection of one clone exclusively before or after weaning or at both time points (as weaning is thought to massively change the intestinal milieu), dominance and solitariness of a clone. Numerical criteria covered the maximum numbers of isolates of one clone in one sample (as we included 20 isolates of one sample there was a theoretical range between 0 and 20 isolates per clone); total numbers including all isolates from all samples of one clone (as we included 800 isolates from all piglets over the whole eight week period and found 44 clones a theoretical range between 1 and 757); numbers of colonized piglets with a specific clone (as we included five piglets a theoretical range between 0 and 5); numbers of time points of the detection of a specific clone (as we sampled over an eight week period a theoretical range between 1 and 8). As there were only very few clones positive, the following resistance phenotypes and genotypes were excluded from detailed statistical analysis: resistance to kanamycin, neomycin and chloramphenicol and the resistance genes *cmlA*, *catA1 *and *aph(3')-Ia*.

There were no significant associations between resistance/resistance genes and the time points of isolation of clones (before or after weaning or at both time points), the dominance of *E. coli *in one sample or the probability to be a solitary clone. Additionally, resistance phenotypes and genotypes did not significantly correlate with the numerical criteria "maximum numbers of isolates of one clone in one sample", "total numbers including all isolates from all samples of one clone", "numbers of colonized piglets with a specific clone" and "numbers of time points of the detection of a specific clone". Thus, resistant clones obviously had no significant advantage or disadvantage in competition with susceptible clones and clones with resistance to several antimicrobial agents had no significant advantage or disadvantage in competition with clones showing resistance to only one or two antimicrobial agents. Clones exhibiting antimicrobial resistance and carrying resistance genes had often even a slightly better colonization success than susceptible clones. Examples are pictured in Table 2. Values of numerical criteria were higher for clones with at least one resistance compared to susceptible clones and values of numerical criteria were higher for clones with resistance to three or more antimicrobial agents compared to clones with less than three resistance properties (Table 2). Clones which exhibited resistance to a specific antimicrobial agent had no significant colonization advantage compared to susceptible clones, but values of all four numerical criteria could also be higher for such resistant clones compared to susceptible clones as seen for ampicillin-resistant clones (Table 3).

**Table 2:** Colonization Study: Comparison of antimicrobial resistance and a colonization success of *E. coli *clones from the rectal content of 5 piglets.

	**resistance to No. of antimicrobial agents per clone**											
	**0**	**1**	**2**	**3**	**4**	**5**	**6**	**7**	**< 3**	**3 ≤**	**0**	**1 ≤**

**number of clones**	**n = 14**	**n = 6**	**n = 8**	**n = 8**	**n = 3**	**n = 3**	**n = 1**	**n = 1**	**n = 28**	**n = 16**	**n = 14**	**n = 30**

maximum numbers in one sample ^a^	1 (1/20)*	2 (1/20)	1.5 (1/15)	2 (1/19)	10 (5/18)	2 (1/9)	1	13	1.5 (1/20)	3.5 (1/19)	1 (1/20)	2 (1/20)
total numbers in all samples ^b^	2 (1/79)	3 (1/106)	3 (1/16)	3 (1/199)	26 (5/41)	2 (1/55)	1	20	2 (1/106)	4.5 (1/199)	2 (1/79)	4 (1/199)
numbers of piglets ^c^	1.5 (1/4)	1.5 (1/5)	1.5 (1/2)	1.5 (1/5)	4 (1/5)	1 (1/5)	1	3	1.5 (1/5)	1.5 (1/5)	1.5 (1/4)	1.5 (1/5)
time points ^d^	1 (1/5)	1.5 (1/4)	1.5 (1/2)	1.5 (1/8)	3 (1/5)	1 (1/6)	1	3	1 (1/5)	1.5 (1/8)	1 (1/5)	1.5 (1/8)

^a ^maximum numbers of isolates of one clone in one sample, as we included 20 isolates of one sample there was a theoretical range between 0 and 20 isolates per clone

^b ^total numbers including all isolates from all samples of one clone, as we included 800 isolates from all piglets over the whole eight week period and detected 44 clones a theoretical range between 1 and 757.

^c ^numbers of colonized piglets with a specific clone, as we included five piglets a theoretical range between 0 and 5

^d ^numbers of time points of the detection of a specific clone, as we sampled over an eight week period a theoretical range between 1 and 8

* median (min/max)

**Table 3:** Colonization Study: Comparison of single antimicrobial resistance and a colonization success of *E. coli *clones from the rectal content of 5 piglets.

	**resistance to**									
	
	**ampicillin**		**streptomycin**		**spectinomycin**		**tetracycline**		**sulfamethoxazole**	
	**yes**	**no**	**yes**	**no**	**yes**	**no**	**yes**	**no**	**yes**	**no**
**number of clones**	**n = 10**	**n = 34**	**n = 13**	**n = 31**	**n = 9**	**n = 35**	**n = 30**	**n = 14**	**n = 15**	**n = 29**

maximum numbers ^a^	11 (1/19)*	2 (1/20)	2 (1/19)	2 (1/20)	2 (1/19)	2 (1/20)	2 (1/20)	1 (1/20)	2 (1/19)	2 (1/20)
total numbers in all samples ^b^	16 (1/199)	2 (1/106)	2 (1/48)	4 (1/199)	4 (1/48)	2 (1/199)	4 (1/199)	2 (1/79)	4 (1/199)	2 (1/106)
numbers of piglets ^c^	2 (1/5)	1 (1/5)	1 (1/5)	2 (1/5)	1 (1/5)	2 (1/5)	1.5 (1/5)	2 (1/4)	1 (1/5)	2 (1/5)
time points ^d^	2 (1/8)	1 (1/6)	1 (1/6)	2 (1/8)	1 (1/6)	1 (1/8)	1.5 (1/8)	1 (1/4)	1 (1/8)	1 (1/6)

^a ^maximum numbers of isolates of one clone in one sample, as we included 20 isolates of one sample there was a theoretical range between 0 and 20 isolates per clone

^b ^total numbers including all isolates from all samples of one clone, as we included 800 isolates from all piglets over the whole eight week period and detected 44 clones a theoretical range between 1 and 757.

^c ^numbers of colonized piglets with a specific clone, as we included five piglets a theoretical range between 0 and 5

^d ^numbers of time points of the detection of a specific clone, as we sampled over an eight week period a theoretical range between 1 and 8

* median (min/max)

### Analysis of dependencies between antimicrobial resistance/resistance genes and ExPEC-typical virulence associated genes (VAGs) (Colonization Study)

As it was recently shown that VAGs typical for extraintestinal pathogenic *E. coli *might play a role for intestinal colonization [16] and thus might play a role for "cross-selection" we analyzed dependencies between antimicrobial resistance/resistance genes and ExPEC-typical VAGs. All clones had been already tested for the presence of 32 VAGs [17] (Figure 1). In our present study we related the occurrence of resistance/resistance genes with the occurrence of VAGs. There were no significant correlations between the absolute number of resistance/resistance genes and the absolute number of VAGs in one clone. Only the occurrence of the group of resistance genes *bla*_TEM _and thus the occurrence of the resistance to ampicillin was positively associated with the absolute numbers of VAGs. Here, ampicillin-resistant *E. coli *clones had significantly more VAGs compared to ampicillin-susceptible *E. coli *clones (p < 0.05). Regarding single resistance genes, the following significant positive associations were observed: the occurrence of *bla*_TEM _was associated with the occurrence of *fyuA*, *irp2*, *iucD*, *sitD *ep., *cva*, *iss*; the occurrence of *tet*(A) was associated with the occurrence of *tsh*; the occurrence of *strA *was associated with the occurrence of *sitD *ep There was one significant negative association: the occurrence of *tet*(B) was negatively associated with the occurrence of *mat*. Here, *tet*(B)-positive clones carried significantly less frequent the gene *mat *compared to *tet*(B)-negative clones (p < 0.05).

### Analysis of associations within antimicrobial resistance and within resistance genes (Resistance Status Study plus Colonization Study)

Both data from the Resistance Status Study as well as from the Colonization Study were pooled. There were significant positive associations between ampicillin and sulfamethoxazole (p < 0.01), spectinomycin and streptomycin (p < 0.001), tetracycline and streptomycin (p < 0.001), tetracycline and spectinomycin (p < 0.01) and tetracycline and sulfamethoxazole (p < 0.005). Additionally, there were significant positive associations between the genes *bla*_TEM _and *sul2 *(p < 0.01) and *tet*(B) and *strA/B *(p < 0.05) and a significant negative association between the genes *tet*(A) and *tet*(B) (p < 0.05).

## Discussion

In this retrospective study we described the antimicrobial resistance status and resistance gene patterns of commensal *E. coli *of one clinical healthy pig production unit and focused on effects of resistance and resistance genes on different nominal and numerical colonization parameters in a small piglet group which were not treated with antimicrobial substances. The aim was to demonstrate possible competition advantages/disadvantages of resistant relative to susceptible bacteria in the porcine intestine. In our study we did not find a significant dependency between one of the nominal and numerical colonization parameters and the total numbers of resistance or resistance genes or between one of the nominal and numerical colonization parameters and a specific resistance or a specific resistance gene. Additionally, resistance and resistance genes were not significantly different in *E. coli *from both the sow and their piglets. Conclusively, resistant *E. coli *were not significantly advantaged or disadvantaged in competition with susceptible *E. coli *and resistance to more agents was not detrimental in comparison to single resistant *E. coli *in the context of their colonization abilities in the fast developing intestine of piglets or after weaning. Notably, resistant bacteria could be isolated at a slightly higher frequency. Hence, we showed that resistant bacteria will not be inevitably outcompeted by susceptible bacteria in the porcine intestine. This might help to understand the occurrence of resistant bacteria in animal populations over long time periods and might substantiate the potential risk of the application of antimicrobial substances in the animal production.

In general, expression of resistance is thought to burden the bacterial metabolism. Consequently, resistant *E. coli *would have a disadvantage in competition with susceptible *E. coli*. This disadvantage might be compensated by other mechanisms which seemed to play an important role also in our study. Aside from an "adaptation of bacteria to the fitness costs of antibiotic resistance" [5,6], "cross-selections" can compensate disadvantages caused by a single gene due to the presence and expression of other genes which are present on the same mobile genetic element. Resistance is often associated with plasmids, transposons and integrons which can also carry genes coding for other antimicrobial resistance or coding for virulence or colonization factors. In fact, it was shown, that extraintestinal *E. coli *(ExPEC) possessed plasmids (e.g. the plasmid pTJ100 with a size of approximately 100 kb) which carried a mosaic of virulence associated genes, insertion sequences, antimicrobial resistance genes, and their remnants. Many of the resistance genes found on the plasmid pTJ100 could be expressed under laboratory conditions [35]. Also in the present study we have shown significant associations between the occurrence of antimicrobial resistance genes and the occurrence of VAGs, between the occurrence of specific antimicrobial resistance genes as well as between the occurrence of several antimicrobial resistance genes indicating that cross-selection might also affect the intestinal colonization success of *E. coli *in piglets. Significant positive associations between antimicrobial resistance genes and VAGs were frequently observed related to iron acquisition genes (*fyuA*, *irp2*, *iucD*, *sitD *ep.). We have previously shown that iron acquisition genes might play a prominent role for the intestinal colonization success in piglets [17]. Conclusively, it might be assumed that the competition disadvantages due to antimicrobial resistance might have been compensated by the presence and expression of iron acquisition genes in such clones in our study.

A second observation of the present study is worth to be discussed. There were many data available about the distribution of resistance in clinical porcine *E. coli *isolates. Such studies focused on the general occurrence and distribution of resistance in pathogenic *E. coli*. Isolates of these studies were mostly not epidemiologically linked since protocols for such sample procedures recommend that susceptibility testing should be done for not more than one isolate per *E. coli *from the same epidemiological unit per year to ensure the representativeness of bacterial isolates [14]. Conclusively, these studies were not able to comprehensively describe the resistance status of the *E. coli *microflora of single pig production units. In contrast, there were only a very limited number of publications available which reported in detail about the resistance status of commensal *E. coli *of single pig production units. It was obvious that these publications of temporal and spatial distant examinations described similar *E. coli *resistance and resistance patterns. Hinton and co-workers in the UK [36], Sunde and co-workers in Norway [37], Alali and co-workers in the U.S.A[38]. and Stannarius and co-workers in Switzerland [39] described above all resistance to ampicillin, chloramphenicol, kanamycin, streptomycin, sulphonamides, and tetracycline with different resistance patterns. In our German study we detected similar resistance and resistance patterns. Conclusively, resistance to ampicillin, chloramphenicol, kanamycin, streptomycin, sulphonamides, and tetracycline are a common and worldwide distributed feature of porcine commensal *E. coli *which seem to reflect the application of these antimicrobial substances over decades in human and animal health and high transmission rates of relevant resistance by mobile genetic elements. Thus, the majority of commensal *E. coli *clones from one pig production unit as well as from a single animal can be resistant. Moreover, if we assume that all resistant clones which were isolated from the five piglets and the bottom of the sow box during our Colonization Study originated from this one brood sow, 49 resistant *E. coli *clones with 17 different resistance profiles and 34 different resistance gene profiles were present in this single animal. Additionally, resistance and resistance genes of clones from this one sow were representative for clones isolated from 15 piglets of the same production unit and thus possible representative for the whole single pig production unit.

## Conclusion

We showed that resistance to ampicillin, chloramphenicol, kanamycin, streptomycin, sulphonamides, and tetracycline were common in commensal porcine intestinal *E. coli *with different combinations of resistance to different antimicrobial agents and a variety of resistance genes. The presence of antimicrobial resistance and the corresponding resistance genes were not associated with a colonization advantage or disadvantage in the intestine of young piglets. The existence of the tested resistance might not play a dominant role for an intestinal colonization success in pigs in the absence of antimicrobial drugs, or cross-selection of other colonization factors e.g. VAGs might compensate "the cost of antibiotic resistance". Results of the present study indicate that resistant strains are not outcompeted by susceptible bacteria in the porcine intestine.

## Abbreviations

The following abbreviations were used: ExPEC: extraintestinal pathogenic *E. coli*; PFGE: pulsed field gel electrophoresis; VAGs: virulence associated genes.

## Competing interests

The authors declare that they have no competing interests.

## Authors' contributions

PS conceived the study, the study design, isolated *E. coli*, carried out the affiliation of isolates to clones, participated in the determination of pheno- and genotypical resistance, interpreted the data and drafted the manuscript. KK carried out specific PCRs for the detection of resistance genes and helped to draft the manuscript. SG carried out large parts of the determinations of pheno- and genotypical resistance. MF performed the statistical analysis. SS participated in the study design, coordinated the determinations of pheno- and genotypical resistance and helped to draft the manuscript. CE carried out PCRs for the detection of ExPEC-typical VAGs. LHW participated in the study design and coordination of the study and helped to draft the manuscript. All authors read and approved the final manuscript.

## Supplementary Material

Additional file 1 **Resistance Status Study. Antimicrobial resistance and resistance genes of 49 *E. coli *clones from clinically healthy domestic piglets of one pig production unit**. The table demonstrates phenotypical and genotypical resistance pattern of all 49 *E. coli *isolates from the Resistance Status Study. Click here for file

Additional file 2 **Colonization Study. Antimicrobial resistance and resistance genes of 68 *E. coli *clones from the rectal content and teats of the sow, the bottom of the box and the piglets**. The table demonstrates phenotypical and genotypical resistance pattern of all 68 *E. coli *isolates from the Colonization Study. Click here for file
