# Supplementary material for: Antimicrobial resistances do not affect colonization parameters of intestinal E. coli in a small piglet group
Source: Gut Pathog. 2009 Oct 8;1:18. doi: 10.1186/1757-4749-1-18 (PMC2766387; doi:10.1186/1757-4749-1-18)
Supplement: Additional file 1 — Resistance Status Study. Antimicrobial resistance and resistance genes of 49 E. coli clones from clinically healthy domestic piglets of one pig production unit. The table demonstrates phenotypical and genotypical resistance pattern of all 49 E. coli isolates from the Resistance Status Study. [file 1757-4749-1-18-S1.DOC]

**Additional file 1: Resistance Status Study. Antimicrobial resistance and resistance genes of 49 *E. coli* clones from clinically healthy domestic piglets of one pig production unit.**

|  |  | resistance and resistance genes | | | | | | | | | | | | | |
| --- | --- | --- | --- | --- | --- | --- | --- | --- | --- | --- | --- | --- | --- | --- | --- |
| resistance to no. of agents | number of strains | AMP | gene | CHL | gene | KAN/  NEO | gene | TET | gene | SMZ | gene | SPT | gene | STR | gene |
|  |  |  |  |  |  |  |  |  |  |  |  |  |  |  |  |
| 7 | 1 | + | *bla*_TEM_ |  |  | + | *aph(3’)-Ia* | + | *tet*(A) | + | *sul2* | + | *aadA* | + | *strA/strB, aadA* |
|  |  |  |  |  |  |  |  |  |  |  |  |  |  |  |  |
| 6 | 1 | + | *bla*_TEM_ |  |  | + | *aph(3’)-Ia* | + | *tet*(A) | + | *sul1, sul2* |  |  | + | *strA/strB* |
|  |  |  |  |  |  |  |  |  |  |  |  |  |  |  |  |
|  | 1 | + | *bla*_TEM_ |  |  |  |  | + | *tet*(A) | + | *sul1, sul2* | + | *aadA* | + | *strA/strB, aadA* |
| 5 | 1 | + | *bla*_TEM_ |  |  |  |  | + | *tet*(A) | + | *sul2* | + | *aadA* | + | *strA/strB, aadA* |
|  | 2 | + | *bla*_TEM_ |  |  |  |  | + | *tet*(A) | + | *sul2* | + | *aadA* | + | *aadA* |
|  | 2 |  |  |  |  | + | *aph(3’)-Ia* | + | *tet*(B) | + | *sul2* |  |  | + | *strA/strB* |
|  |  |  |  |  |  |  |  |  |  |  |  |  |  |  |  |
|  | 2 | + | *bla*_TEM_ |  |  |  |  | + | *tet*(A) | + | *sul2* |  |  | + | *strA/strB* |
|  | 1 |  |  | + | *catA1* |  |  | + | *tet*(A) |  |  | + | *aadA* | + | *aadA* |
| 4 | 3 |  |  |  |  |  |  | + | *tet*(A) | + | *sul1* | + | *aadA* | + | *aadA* |
|  | 1 |  |  |  |  |  |  | + | *tet*(A) | + | *sul2* | + | *aadA* | + | *aadA* |
|  | 1 |  |  |  |  |  |  | + | *tet*(A) | + | *sul3* | + | *aadA* | + | *aadA* |
|  |  |  |  |  |  |  |  |  |  |  |  |  |  |  |  |
| 3 | 1 | + | *bla*_TEM_ |  |  |  |  | + | *tet*(B) |  |  |  |  | + | *strA/strB* |
|  | 4 |  |  |  |  |  |  | + | *tet*(A) |  |  | + | *aadA* | + | *aadA* |
|  |  |  |  |  |  |  |  |  |  |  |  |  |  |  |  |
|  | 1 |  |  |  |  |  |  |  |  |  |  | + | *aadA* | + | *aadA* |
| 2 | 2 |  |  |  |  |  |  | + | *tet*(B) |  |  |  |  | + | *strA/strB* |
|  | 1 |  |  |  |  |  |  | + | *tet*(A) | + | unknown |  |  |  |  |
|  | 1 |  |  |  |  |  |  | + | *tet*(A) | + | *sul2* |  |  |  |  |
|  |  |  |  |  |  |  |  |  |  |  |  |  |  |  |  |
| 1 | 6 |  |  |  |  |  |  | + | *tet*(A) |  |  |  |  |  |  |
|  |  |  |  |  |  |  |  |  |  |  |  |  |  |  |  |
| 0 | 16 |  |  |  |  |  |  |  |  |  |  |  |  |  |  |

+ resistant to this antimicrobial agent

AMP: ampicillin, CHL: chloramphenicol, KAN: kanamycin, NEO: neomycin, TET: tetracycline, SMZ: sulfamethoxazole, SPT: spectinomycin, STR: streptomycin
