# Supplementary material for: Antimicrobial resistances do not affect colonization parameters of intestinal E. coli in a small piglet group
Source: Gut Pathog. 2009 Oct 8;1:18. doi: 10.1186/1757-4749-1-18 (PMC2766387; doi:10.1186/1757-4749-1-18)
Supplement: Additional file 2 — Colonization Study. Antimicrobial resistance and resistance genes of 68 E. coli clones from the rectal content and teats of the sow, the bottom of the box and the piglets. The table demonstrates phenotypical and genotypical resistance pattern of all 68 E. coli isolates from the Colonization Study. [file 1757-4749-1-18-S2.DOC]

**Additional file 2: Colonization Study. Antimicrobial resistance and resistance genes of 68 *E. coli* clones from the rectal content and teats of the sow, the bottom of the box and the piglets.**

|  |  | resistance and resistance genes | | | | | | | | | | | | | |
| --- | --- | --- | --- | --- | --- | --- | --- | --- | --- | --- | --- | --- | --- | --- | --- |
| resistance to no. of agents | number of strains | AMP | gene | CHL | gene | KAN/NEO | gene | TET | gene | SMZ | gene | SPT | gene | STR | gene |
|  |  |  |  |  |  |  |  |  |  |  |  |  |  |  |  |
| 7 | 1 | + | *bla*_TEM_ |  |  | + | *aph(3’)-Ia* | + | *tet*(A) | + | *sul2* | + | *aadA* | + | *strA/strB, aadA* |
|  |  |  |  |  |  |  |  |  |  |  |  |  |  |  |  |
| 6 | 1 | + | *bla*_TEM_ |  |  | + | *aph(3’)-Ia* | + | *tet*(A) | + | *sul1* |  |  | + | *strA/strB* |
|  |  |  |  |  |  |  |  |  |  |  |  |  |  |  |  |
|  | 1 | + | *bla*_TEM_ |  |  | + | *aph(3’)-Ia* | + | *tet*(A) | + | *sul2, sul3* |  |  |  |  |
| 5 | 1 | + | *bla*_TEM_ |  |  |  |  | + | *tet*(B) | + | *sul2* | + | *unknown* | + | *strA/strB* |
|  | 1 | + | *bla*_TEM_ |  |  |  |  | + | *tet*(B) | + | *sul2* | + | *aadA* | + | *strA/strB, aadA* |
|  | 1 |  |  | + | *cmlA/B* |  |  | + | *tet*(A) | + | *sul3* | + | *aadA* | + | *aadA* |
|  |  |  |  |  |  |  |  |  |  |  |  |  |  |  |  |
|  | 1 | + | *bla*_TEM_ |  |  |  |  | + | *tet*(B) | + | unknown |  |  | + | *strA/strB* |
| 4 | 1 |  |  | + | *catA1* |  |  | + | *tet*(A) |  |  | + | *aadA* | + | *aadA* |
|  | 1 |  |  |  |  |  |  | + | *tet*(B) | + | unknown | + | *aadA* | + | *aadA* |
|  | 1 |  |  |  |  |  |  | + | *tet*(A) | + | *sul2* | + | *aadA* | + | *aadA* |
|  |  |  |  |  |  |  |  |  |  |  |  |  |  |  |  |
|  | 1 | + | *bla*_TEM_ | + | *cmlA/B* |  |  |  |  | + | *sul3* |  |  |  |  |
|  | 1 | + | *bla*_TEM_ |  |  |  |  | + | *tet*(A) | + | *sul1, sul2* |  |  |  |  |
|  | 1 | + | *bla*_TEM_ |  |  |  |  | + | *tet*(A) | + | *sul2, sul3* |  |  |  |  |
|  | 1 |  |  | + | *cmlA/B* |  |  | + | *tet*(A) | + | *sul3* |  |  |  |  |
| 3 | 1 |  |  | + | *catA1* |  |  | + | *tet*(A) | + | *sul2* |  |  |  |  |
|  | 2 |  |  |  |  |  |  | + | *tet*(B) |  |  | + | *aadA* | + | *strA/strB, aadA* |
|  | 1 |  |  |  |  |  |  | + | *tet*(B) |  |  | + | *aadA* | + | *aadA* |
|  | 2 |  |  |  |  |  |  | + | *tet*(A) |  |  | + | *aadA* | + | *strA/strB, aadA* |
|  | 2 |  |  |  |  |  |  | + | *tet*(A) |  |  | + | *aadA* | + | *aadA* |
|  | 1 |  |  |  |  |  |  | + | *tet*(B) | + | *unknown* |  |  | + | *strA/strB* |
|  |  |  |  |  |  |  |  |  |  |  |  |  |  |  |  |
|  | 2 | + | *bla*_TEM_ |  |  |  |  | + | *tet*(A) |  |  |  |  |  |  |
|  | 2 | + | *bla*_TEM_ |  |  |  |  | + | *tet*(B) |  |  |  |  |  |  |
|  | 2 |  |  |  |  |  |  | + | *tet*(B) |  |  |  |  | + | *strA/strB* |
|  | 3 |  |  |  |  |  |  | + | *tet*(A) |  |  |  |  | + | *strA/strB* |
| 2 | 2 |  |  |  |  |  |  | + | *tet*(A) | + | *sul1* |  |  |  |  |
|  | 1 |  |  |  |  |  |  | + | *tet*(A) | + | *sul2* |  |  |  |  |
|  | 3 |  |  |  |  |  |  | + | *tet*(B) | + | *sul2* |  |  |  |  |
|  | 1 |  |  |  |  |  |  | + | *tet*(A) |  | unknown |  |  |  |  |
|  | 1 |  |  |  |  |  |  | + | *tet*(B) |  | unknown |  |  |  |  |
|  |  |  |  |  |  |  |  |  |  |  |  |  |  |  |  |
| 1 | 8 |  |  |  |  |  |  | + | *tet*(A) |  |  |  |  |  |  |
|  | 1 |  |  |  |  |  |  | + | *tet*(B) |  |  |  |  |  |  |
|  |  |  |  |  |  |  |  |  |  |  |  |  |  |  |  |
| 0 | 19 |  |  |  |  |  |  |  |  |  |  |  |  |  |  |

+ resistant to this antimicrobial agent

AMP: ampicillin, CHL: chloramphenicol, KAN: kanamycin, NEO: neomycin, TET: tetracycline, SMZ: sulfamethoxazole, SPT: spectinomycin, STR: streptomycin
